# A Mid-Life Vitamin A Supplementation Prevents Age-Related Spatial Memory Deficits and Hippocampal Neurogenesis Alterations through CRABP-I

**DOI:** 10.1371/journal.pone.0072101

**Published:** 2013-08-19

**Authors:** Katia Touyarot, Damien Bonhomme, Pascale Roux, Serge Alfos, Pauline Lafenêtre, Emmanuel Richard, Paul Higueret, Véronique Pallet

**Affiliations:** 1 Univ. Bordeaux, Nutrition et Neurobiologie Intégrée, UMR 1286, Bordeaux, France; 2 INRA, Nutrition et Neurobiologie Intégrée, UMR 1286, Bordeaux, France; 3 INSERM, Transfert de gènes à visée thérapeuthique dans les cellules souches, U1035, Bordeaux, France; VIB & Katholieke Universiteit Leuven, Belgium

## Abstract

Age-related memory decline including spatial reference memory is considered to begin at middle-age and coincides with reduced adult hippocampal neurogenesis. Moreover, a dysfunction of vitamin A hippocampal signalling pathway has been involved in the appearance of age-related memory deficits but also in adult hippocampal neurogenesis alterations. The present study aims at testing the hypothesis that a mid-life vitamin A supplementation would be a successful strategy to prevent age-related memory deficits. Thus, middle-aged Wistar rats were submitted to a vitamin A enriched diet and were tested 4 months later in a spatial memory task. In order to better understand the potential mechanisms mediating the effects of vitamin A supplementation on hippocampal functions, we studied different aspects of hippocampal adult neurogenesis and evaluated hippocampal CRABP-I expression, known to modulate differentiation processes. Here, we show that vitamin A supplementation from middle-age enhances spatial memory and improves the dendritic arborisation of newborn immature neurons probably resulting in a better survival and neuronal differentiation in aged rats. Moreover, our results suggest that hippocampal CRABP-I expression which controls the intracellular availability of retinoic acid (RA), may be an important regulator of neuronal differentiation processes in the aged hippocampus. Thus, vitamin A supplementation from middle-age could be a good strategy to maintain hippocampal plasticity and functions.

## Introduction

The vitamin A, through its main metabolite retinoic acid (RA), plays a key role in brain development by regulating neuronal differentiation, neurite outgrowth and the anteroposterior axis of the neural tube [1], [2], [3]. It is now established that retinoids are required for cognitive functions in the adulthood [4], [5], [6], [7] and that retinoid hyposignalling contributes to the deterioration of hippocampal synaptic plasticity and functions [8], [9], [10]. In aged rodents, the naturally occurring hypoactivity of the retinoid signalling pathway has been associated with the reduction of hippocampal synaptic plasticity [11], [12], known to underlie at least in part relational memory processing [13], [14]. Indeed, pharmacological activation of retinoid signalling by short-term RA treatment in aged mice restored their impaired hippocampal long-term potentiation as well as their long-term relational memory deficits [7]. Moreover, it has recently been shown that a lifelong nutritional vitamin A supplementation induced similar (even more) beneficial effects on hippocampal plasticity and memory processes [15].

One hippocampal plasticity that has mainly been studied in the recent decades is adult neurogenesis. Indeed, new neurons can be generated and survive in the adult dentate gyrus (cell proliferation, survival mechanisms and subsequent differentiation processes) but the neurogenenic rate declines precipitously from middle-age [16], [17], [18], [19], [20]. These newly born neurons have been shown to be preferentially recruited into circuits supporting various types of learning and memory [21], [22], [23], [24], [25], [26], providing evidence for a critical role of adult neurogenesis in hippocampus-dependent memory including spatial memory in the Morris Water Maze [27], [28], [29], [30]. It has been reported that decreased neurogenesis correlates with aging-associated impairments in learning and memory [31], [32] but some controversial studies have shown that poorer performance was associated to a better neuronal differentiation and survival [33], [34].

Vitamin A and its derivatives, as RA, act on memory processes by modulating different aspects of hippocampal plasticity including synaptic plasticity [7], [12] but also adult neurogenesis in the dentate gyrus (DG) [35], [36], [37], [38]. Indeed, it has recently been demonstrated that RA treatment can restore normal level of neurogenesis in vitamin A deficient rats [36]. Moreover, a gradient of RA, generated in the meninges, would differentially modulate adult neurogenesis between the two pyramidal blades of the rodent DG [38]. However, no studies have explored the possibility to stimulate adult hippocampal neurogenesis by acting on the retinoid signalling pathway during senescence. It is now accepted, that in rodents and humans, aging induces an alteration of retinol metabolism that leads to a reduction of cellular RA bioavailability in target tissues [12], [39], [40]. In the brain, the bioavailability of RA is controlled, from circulating retinol, by local RA anabolizing and catabolizing enzymes, but also by RA binding proteins such as CRABP-I (cellular retinoic acid binding protein I) that is highly expressed in the DG [38], [41]. The crucial role of CRABP-I in neurogenesis has previously been revealed during development [42]. Interestingly, a surexpression of CRABP-I has been associated with a reduction of differentiation in human neuroblastoma cells suggesting that the regulation of RA bioavailability by CRABP-I could be determinant in the modulation of neurogenesis [43]. However, its potential role in adult hippocampal neurogenesis has not been elucidated yet.

In the present study, we consequently investigated the ability of a vitamin A supplementation started at middle age, to counteract age-related spatial reference memory decline. In order to better understand the potential mechanisms mediating the effects of vitamin A supplementation on hippocampal functions, we studied different aspects of hippocampal neurogenesis (cell proliferation, cell survival and neuronal differentiation processes). Besides, we measured plasma retinol levels and hippocampal CRABP-I mRNA expression. In this study, we demonstrated for the first time that a mid-life vitamin A supplementation would be a successful strategy to prevent age-related spatial memory deficits by stimulating the differentiation of newly formed neurons and cell survival in aged rats. Moreover, our results suggest that the control of the cellular bioavailability of RA by CRABP-I could be an essential mechanism in the modulation of hippocampal adult neurogenesis by RA.

## Materials and Methods

### Animals

Male Wistar rats were purchased from Janvier (Le Genest Saint-Isle, France) at the age of weaning (3 weeks) and 13 months (middle-aged). They were housed two per cage in a room with a constant airflow system, controlled temperature (21–23°C), and a 12 h light/dark cycle. They were randomly divided into three experimental groups designed as young and aged groups receiving a control diet (5 month-old and 17 month-old respectively), and enriched-aged group receiving a vitamin A enriched diet (17 month-old). All animals were individually housed from 3 weeks prior to the beginning of the behavioral test until sacrifice. All experiments were performed in accordance with the European Communities Council Directives (86/609/EEC) and the French national Committee (87/848) recommendations. All animal procedures were approved by the Animal Care and Use Committee of Bordeaux.

### Diet

The weaned rats (n = 10) and half of the middle-aged rats (n = 10) were fed with a control diet containing 5 IU retinol/g during 4 months (control condition): they are referred to as the 5-month-old young rats and the 17-month-old aged rats, respectively. The second half of the middle-aged group (n = 10) received the vitamin A-enriched diet (45 IU retinol/g) for the same duration : they are referred to as the 17-month-old enriched-aged rats. The composition of the control diet was the same as the vitamin A-enriched diet, excepted for retinol content. The 45 IU diet was chosen on the basis of literature as a moderate supplementation to avoid potential toxicity of hypervitaminosis (for review, see [44]). Moreover, this vitamin A supplemented diet was shown to counteract declarative-like memory deficits in aged mice when started at the age of 2 months [15].

### Experimental Design

**Figure 1:**
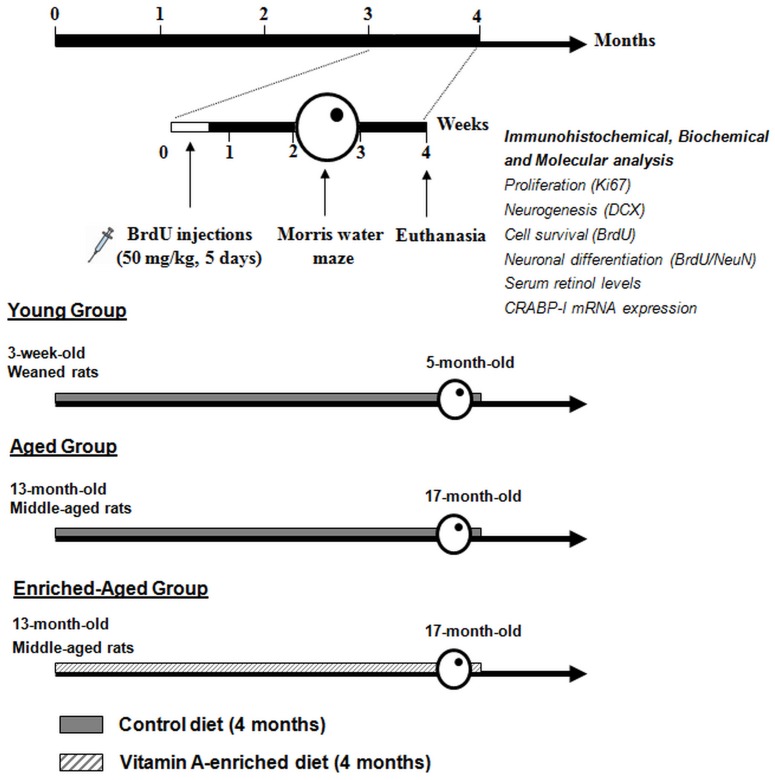
Experimental protocol. Effects of 4 months of vitamin A supplementation started at mid-age on spatial memory, hippocampal neurogenesis and retinoid status in rats. The weaned rats and half of the middle-aged rats were fed with a control diet containing 5 IU retinol/g during 4 months (control condition): they are referred to as the 5-month-old young rats and the 17-month-old aged rats, respectively. The second half of the middle-aged group received the vitamin A-enriched diet (45 IU retinol/g) for the same duration : they are referred to as the 17-month-old enriched-aged rats.

We have studied the effects of a mid-life vitamin A supplementation on a spatial reference memory task and hippocampal neurogenesis, known to be altered during aging (Fig. 1). Thus, the weaned rats (3-week-old) and middle-aged rats (13-month-old) received a control diet or a vitamin A enriched diet during 4 months. 3 months after the beginning of the diets, the three groups (young n = 10, aged n = 10, enriched-aged n = 10) were injected with BrdU for 5 days. Rats were allowed to survive for another three weeks after the last injection of BrdU and continued diet in their respective experimental conditions. All groups were tested in a spatial reference memory task in the water maze and sacrificed one week after the completion of behavioral testing, to analyze hippocampal neurogenesis, serum retinol concentration and mRNA expression of CRABP-I.

### 5-Bromo-2′-deoxyuridine (BrdU) Injections

In order to label the newly born cells and examine hippocampal cell survival and differentiation, BrdU, a thymidine analogue incorporated into genetic material during synthetic DNA phase of mitotic division, was used. Rats received a daily intraperitoneal injection of BrdU (50 mg/kg, Sigma), dissolved in phosphate buffer (0.1 M, pH 8.4), during the 5 consecutive days beginning 3 months after the different diets.

### Behavioral Testing

Rats were tested in a Morris water maze (180 cm in diameter, 60 cm high) filled with water (22°C) made opaque by addition of white paint. An escape platform was hidden 2 cm below the surface of the water in a fixed location in one of four quadrants halfway between the wall and the middle of the pool. Before the start of the training, animals were habituated to the pool without a platform for 1 min/day for 2 days. During training, animals were required to locate the submerged platform by using distal extramaze cues. They were trained for four trials per day (90 s with an intertrial interval of 60 s, starting from three different start points randomized every day) for 9 consecutive days. The swim speed, the time to reach the platform (latency) and the distance covered to find the platform were measured with a computerized tracking system (Videotrack, Viewpoint). On day 10, animals were tested for 60 s in the pool without the platform (probe test). Performance was evaluated by the percentage of time spent in the quadrant where the platform was located during training (target quadrant). Finally, in order to control for visual acuity deficits, the hidden platform was replaced by a visible platform located in the opposite quadrant, and animals were tested for three trials (90 s) over one day. Two aged rats (receiving the control diet and the enriched diet) were excluded from the experiment due to failure to search for the platform during the acquisition phase (floating) leading to a number of 9 animals in the aged group and in the enriched-aged group. For the statistical analyses, results from blocks of trials for each day were averaged for every rat.

### Immunohistochemistry

Rats were euthanized with isoflurane and the brain quickly removed. Half of the dissected brains were washed with 0.9% sodium chloride, followed by 4% paraformaldehyde. After a 3-week postfixation period in paraformaldehyde, 50 µm frontal sections were cut on a vibratome (Leica). Free-floating sections were processed with a standard immunohistochemical procedure [45]. A one–in-ten section was treated for Ki-67 immunoreactivity using a mouse anti-Ki-67 monoclonal antibody (1∶200, Novocastra) or for doublecortin (DCX) immunoreactivity using a goat polyclonal antibody (1∶1000, Santa Cruz Biotechnology). The sections were then incubated with biotinylated secondary horse anti-mouse or donkey anti-goat antibodies respectively (1∶200, AbCys; 1∶200, Amersham). For BrdU labeling, adjacent sections were treated with 2N HCl to denature DNA (30 min at 37°C) and then washed in phosphate buffer. Sections were incubated with a mouse monoclonal anti-BrdU antibody (1∶200, Dako) followed by the biotinylated horse anti-mouse antibody (1∶200, AbCys). Sections were processed in parallel, and immunoreactivities were visualized by the biotin-streptavidin technique (ABC kit, Dako) by using 3,3′-diaminobenzidine as chromogen.

The number of immunoreactive (IR) cells in the left DG was estimated by using a modified version of the optical fractionator method with a systematic random sampling of every 10 sections along the rostrocaudal axis of the DG. On each section, IR cells in the granular and subgranular layers of the DG were counted with a 100x microscope objective. All results are expressed as the total number of cells in the whole DG. The fraction of DCX-IR new neurons with large vertical dendrites (mature new neurons) corresponding to a higher level of differentiation was also counted.

To analyze the phenotype of BrdU labeled cells, 4 rats per group were randomly selected. One in-ten section was incubated with a rat anti-BrdU monoclonal antibody (1∶500, Servibio), which were revealed by using goat cy3-labeled anti-rat IgG antibodies (1∶1000, Interchim). Sections were then incubated with mouse monoclonal anti-NeuN antibodies (1∶1000, Millipore), and bound anti-NeuN monoclonal antibodies were visualized with an Alexa 488 goat anti-rabbit IgG (1∶1000, Interchim). The percentage of BrdU-labeled cells that expressed NeuN was determined throughout the DG by using a fluorescence microscope (Nikon eclipse E400) equipped with Hamamatsu digital camera and piezoelectric z-axis controller. All BrdU double labeled cells were examined, and sections were optically sliced in the Z plane by using a 1 µm interval. Cells were rotated in orthogonal planes to verify double labeling and images were analyzed by using a Nikon NIS-elements acquisition software.

### Real-Time PCR Analysis of CRABP-I Expression

After the removal of the brain, the other half of the hippocampus was rapidly removed and stored at –80°C in order to measure CRABP-I expression. Extraction of RNA was conducted using an extraction kit (TRIzol reagent, Invitrogen) according to the manufacturer’s instructions. The quality and the concentration of RNA were determined by using a nanodrop (ND-1000) (Labtech). Then, the integrity of the purified RNA was verified using the RNA 6000 Nano LabChip kit in combination with the 2100 bioanalyser (Agilent Technologies). Using OligodT and random primers (Promega), cDNA was synthesized with ImPromII reverse transcriptase (Promega). Briefly, 1 µg of total RNA mixed with RNasin (Promega) and DNase (Roche) was incubated at 37°C. Then, OligodT and random primers were added for incubation at 70°C. The reverse transcriptase reaction was performed at 42°C for 60 min in a final volume of 20 µl.

The real-time PCR was performed using the LightCycler 480 system with a 96-well format (Roche Diagnostics) in a final volume of 20 µl, containing 1×LightCycler 480 SYBR Green I Master solution, 0.5 µM of each primer and 6 µl of cDNA. The forward and reverse primer sequences for CRABP-I and Ppib that was used as a house-keeping gene are given in Table 1. The following program started with an initial denaturation step for 10 min at 95°C, then an amplification for 40 cycles (10 s denaturation at 95°C, 6 s annealing at 62°C, and 10 s extension at 72°C), finally a melting curve analysis was run. In order to verify the specificity and the identity of the amplified products : (1) the melting curve analysis showed a single melting peak after amplification, and (2) amplified products for each gene were verified by sequencing with the Big Dye Terminator v1.1. (Applied Biosystems) and analyzed on a ABI3130 sequencer (Applied Biosystems).

**Table 1:** Primers used for Light Cycler RT-PCR.

Gene name	Nucleotide sequence 5′-3′	Productlength (bp)
Ppib	F : GTTCTGGAAGGCATGGATGT	153
	R : TCCCCGAGGCTCTCTCTACT	
CRABP-I	F : TCTTTCCTCCACACACCTCTCC	124
	R : TCATGCAGATGCCAAACCAG	

Sequences are shown for foward (F) and reverse (R) primers. Ppib: peptidylprolyl isomerase B (cyclophilin B); CRABP I : cellular retinoic acid binding protein I.

The peptidylprolyl isomerase B (Ppib) housekeeping gene was used as the reference gene for relative quantification as its expression level was unaffected by our experimental conditions. Quantification data were analyzed using the LightCycler 480 Relative Quantification software (version 1.5). In order to compensate for differences in target and reference gene amplification efficiency, either within or between experiments, this software provides a calibrator-normalized relative quantification including a PCR efficiency correction. Therefore, the results are expressed as the target/reference ratio divided by the target/reference ratio of the calibrator. In our case, the calibrator was chosen among the young rats.

### Measurement of Serum Retinol Concentration

Blood was collected at the sacrifice and spun at 1500 g for 15 minutes. The supernatant was removed and stored at −20°C. Serum retinol was assayed by HPLC according to a previously described method [46].

### Statistical Analysis

Spatial learning and swim speed data were analyzed using a 2-way ANOVA (3 groups) followed by a post-hoc Bonferroni test. Probe test, immunohistochemical (Ki-67 and DCX only) and PCR data were analyzed using a 1-way ANOVA followed by a post-hoc Bonferroni test. Two pairwise comparisons were performed between young and aged rats (age effect) but also between aged and enriched-aged rats (supplementation effect) using Bonferroni adjusted significance levels of 0.025. Probe test comparisons to chance level (25%) were performed in each group using one sample test. Results are expressed as mean and standard error of the mean, and they were considered significant when p<0.025 for ANOVAs and Bonferroni tests or p<0.05 for one sample test.

## Results

### Effects of a Mid-life Vitamin A Supplementation on Spatial Reference Learning and Memory in Aged Rats

The aim of this study was to determine whether a mid-life vitamin A supplementation would be efficient to prevent the age-related spatial memory deficits in rats.

#### Spatial learning

Results from blocks of trials for each training day were averaged for every rat. The latency to reach the hidden platform, the classical parameter used in the Morris water maze task, may reflect differences in the swimming speed of young versus aged rats. Even though we could not really put them on the fore, we rather used the distance covered to reach the platform as a more appropriate measure and as a good index of the acquisition rate for spatial learning. Indeed, the swimming speed displayed by rats over the nine training sessions has been controlled (young rats : 22.6±0.36 cm/s, aged rats: 19.1±0.5 cm/s, enriched-aged rats : 20.9±0.38 cm/s) and the ANOVA revealed no significant difference between groups [F(2,25) = 2.64, p = 0.09] indicating that aged rats did not exhibit motor impairments.

**Figure 2:**
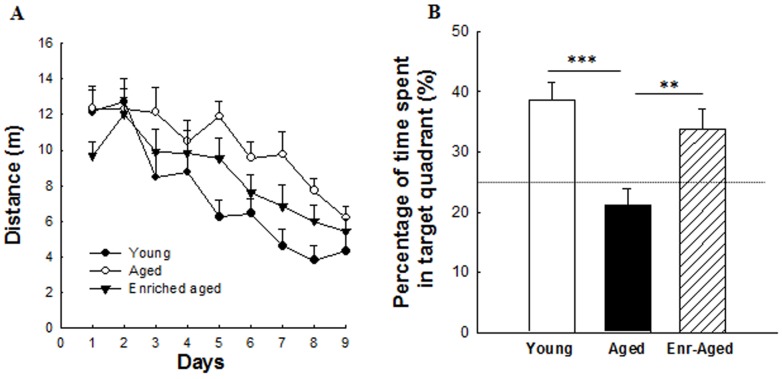
Effects of aging and a mid-life vitamin A supplementation on spatial learning and memory abilities in the Morris water maze. (A) Distance covered by rats to find the hidden platform along nine consecutive days (*Training*; blocks of trials for each training day are averaged) and (B) the recall of the platform location in the target quadrant (probe test) were evaluated in all groups. The dotted line corresponds to chance level (25%). Delayed rate of acquisition and reduced percentage of time in the target quadrant were observed in aged rats. Enriched-aged rats showed a clear spatial reference memory improvement. Significant p values after Bonferroni correction for pairwise comparisons (level α = 0.025; **p<0.01; ***p<0.001).

Young and aged animals learned this task as shown by the progressive decrease in the distance covered to reach the hidden platform over the nine days of training [F(8,200) = 19.49, p<0.001]. A significant group effect [F(2,25) = 4.52, p = 0.02] but without interaction group×days [F(8,200) = 1.54, n.s] was observed in the distance covered by rats to reach the platform (Fig. 2A). Indeed, aged rats travelled significantly longer distance to find the hidden platform than young rats, putting on the fore learning impairments in aged rats (Bonferroni test between aged rats : 10.26±0.4 m and young rats: 7.5±0.5 m, p<0.01). However, this spatial learning deficit was not reversed in aged rats receiving vitamin A enriched diet even if supplemented rats tended to travel a smaller distance to find the platform over the nine training sessions compared to aged rats receiving a control diet (Bonferroni test between aged rats : 10.26**±**0.4 m and enriched-aged rats : 8.5±0.39 m, p = 0.08, n.s).

#### Spatial reference memory

One day later, spatial memory for the platform location was tested using a probe test i.e. in the absence of the platform. ANOVA was performed on the percentage of time spent in the target quadrant in which the platform was placed at training and revealed a group effect [F(2,25) = 8.9, p<0.01]. During the probe test, young rats spent 38.6±2.9% of their time in the target quadrant suggesting that they do remember where the platform was (Fig. 2B). Indeed, one sample test analysis revealed that this percentage is significantly different from the chance level (vs 25%, p = 0.001). On the contrary, aged rats failed to display a memory for the platform location, as indicated by a percentage of time in the target quadrant around the chance level (21.2±2.6% vs 25%, p = 0.2) showing spatial reference memory deficits (Bonferroni test, between aged and young rats, p<0.001). Interestingly, supplemented aged rats spent significantly more time (33.8±3.3%, which is different from the chance level, p = 0.029) than aged rats looking after the platform in the correct quadrant (Bonferroni test, between aged and enriched-aged rats, p<0.01).

#### Visible platform

After the probe trial, on day 11, rats were trained to swim towards a visible platform over three trials for a day. The distance covered to locate the visible platform was similar for all groups [F(2,25) = 1.44, p = 0.25] without interaction group×trials [F(2,50) = 0.82, p = 0.5] indicating that the old subjects did not have any potential visual impairments (data not shown).

### Effects of a Mid-life Vitamin A Supplementation on Hippocampal Neurogenesis in Aged Rats

The effects of retinoids on spatial memory have been proposed to be mediated, at least in part, by a modulation of hippocampal neurogenesis [36]. The aim of this study was thus to determine for the first time, the ability of a mid-life vitamin A supplementation to modulate the three different aspects of hippocampal neurogenesis (cell proliferation, survival of newborn cells, neuronal differentiation processes) during aging.

#### Cell proliferation

**Figure 3:**
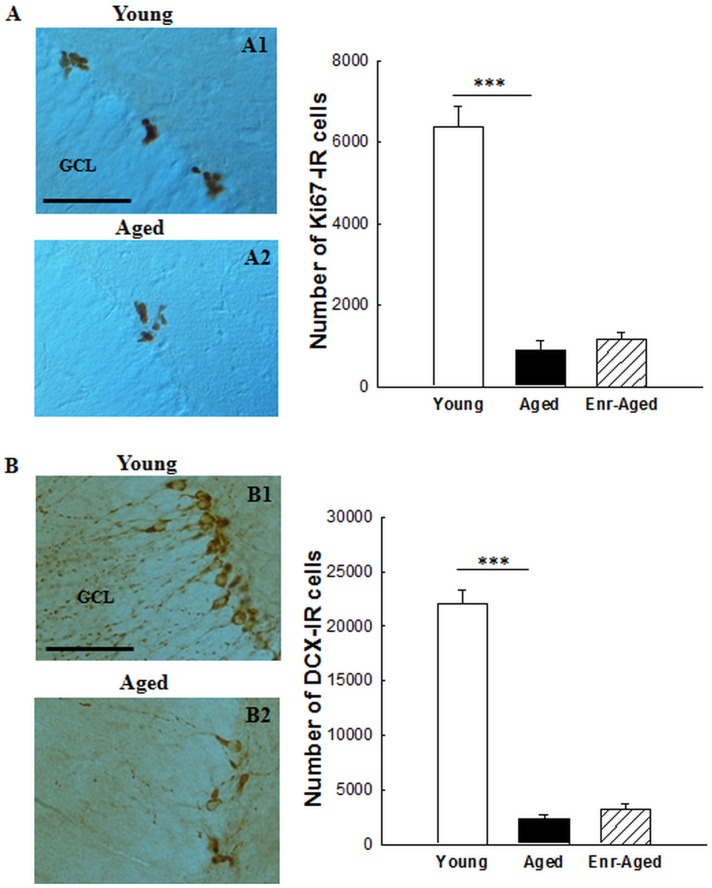
Effects of aging and a mid-life vitamin A supplementation on the number of Ki67-IR and DCX-IR cells. Images show the immunoperoxidase staining of (A) Ki67-IR and (B) DCX-IR cells in the DG granule cell layer of young and aged rats. The graphs indicate the strong reduction of the number of Ki67-IR and DCX-IR cells in aged rats. Vitamin A supplementation does not affect cell proliferation nor the number of immature neurons in aged rats. Significant p values after Bonferroni correction for pairwise comparisons (level α = 0.025; ***p<0.001). Scale bar : 50 µm. GCL = granule cell layer.

Cell proliferation was measured using an endogenous marker of cell cycle, Ki-67. Ki-67-labeled cells were located within the subgranular zone and were isolated or grouped in clusters (Fig. 3A). A quantitative analysis revealed a group effect [F(2,25) = 73.6, p<0.001]. Aging is associated with a significant reduction (−86%) in cell proliferation (Fig. 3B, Bonferroni test, number of Ki-67-IR cells in young rats : 6368±523.7 vs in aged rats : 897.7±224.6, p<0.0001). We tested whether the modulation of retinoid signalling in aged rats could be accompanied by a beneficial effect on cell proliferation. No effect of a mid-life vitamin A supplementation was observed on cell proliferation during aging (Bonferroni test, number of Ki-67-IR cells in aged rats 897.7±224.6 vs in enriched-aged rats : 1153.3±199.2, n.s).

#### Number of immature neurons

The strong reduction in cell proliferation in aged rats suggested a decrease in hippocampal neurogenesis. To test this hypothesis, we quantified the number of immature neurons. We used doublecortin (DCX), a microtubule-associated phosphoprotein, as a marker of neurogenesis. DCX-IR cells were located in the deepest region of granule cell layer (GCL) at the interface with the hilus. In young animals, we observed many DCX-IR cells by contrast to old animals exhibiting a strong reduction in the number of DCX-IR cells (Fig. 3B). Indeed, a quantitative analysis revealed a group effect [F(2,25) = 191.7, p<0.001] and Bonferroni test (p<0.0001) confirmed that aging is associated with a strong reduction (−90%) in the number of immature neurons in the rat hippocampus [Fig. 3B, number of immature neurons in young rats : 22090±1205 vs in aged rats : 2368.8±279.3]. However, vitamin A supplementation had no effect on the number of newly generated immature neurons in aged rats (Bonferroni test, n.s, number of immature neurons in enriched-aged rats : 3175.5±530.4).

#### Dendritic arborisation of immature neurons, cell survival and neuronal differentiation

**Figure 4:**
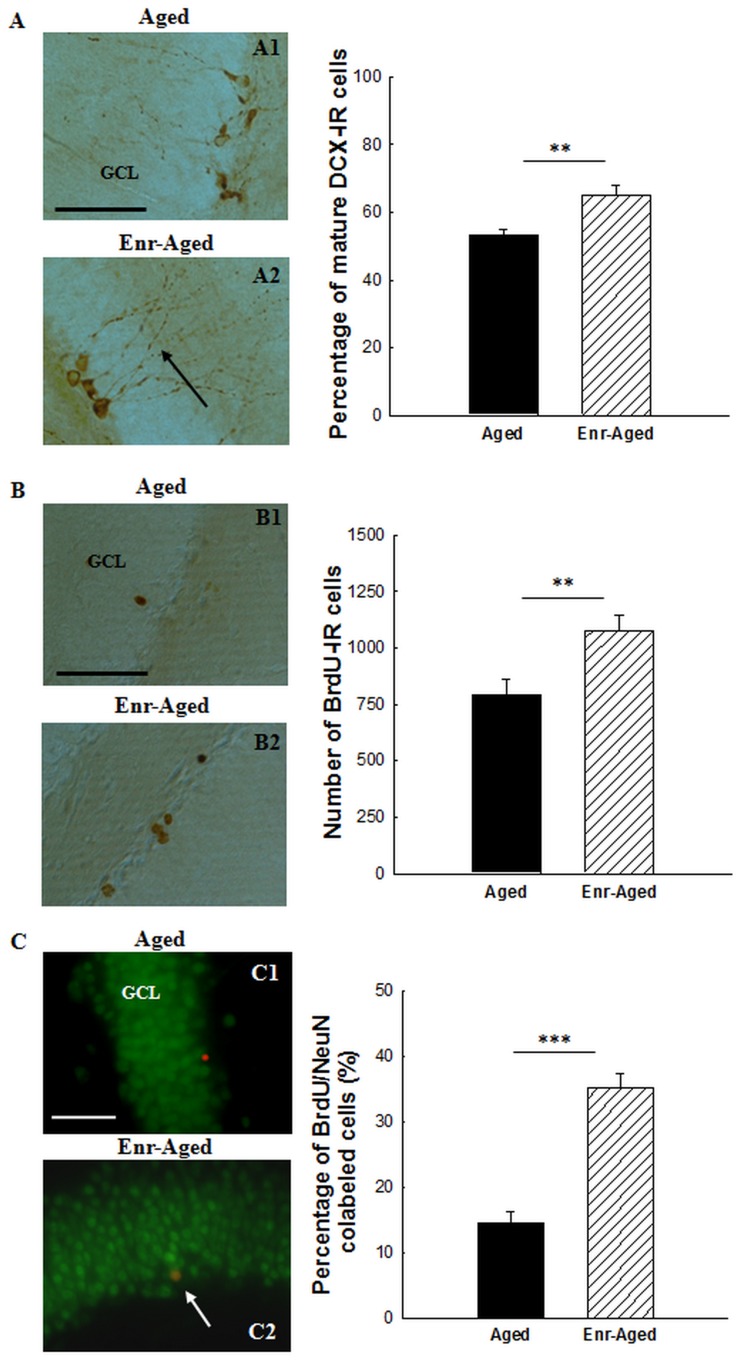
Effects of a mid-life vitamin A supplementation on maturation of DCX-IR cells, survival of BrdU-IR cells and neuronal differentiation in aged rats. Images show immunostaining of (A) DCX-IR cells (B) BrdU-IR cells (C) BrdU/NeuN colabeled cells in the DG granule cell layer of aged and enriched-aged rats. Enriched-aged rats showed (A) an increased number of mature DCX-IR cells, (B) an increased number of BrdU-IR cells and (C) a higher percentage of BrdU/NeuN colabeled cells. Significant p values after ANOVA (**p<0.01,***p<0.001). The black arrow in A2 points to DCX-IR cells with vertical dendritic ramifications and the white arrow in C2 to a BrdU/NeuN colabeled cell in enriched aged rats. Scale bar : 50 µm. GCL = granule cell layer.

Immunohistochemical images revealed that DCX-positive immature neurons in aged rats were not only fewer in number but also characterized by a decrease in dendritic arborisation, potentially reflecting retarded maturation (Fig. 4A). Therefore, we investigated the degree of maturation of the newly born neurons by differentiating more mature DCX-IR cells with vertical dendrites emanating from the soma and extending into the dentate molecular layer through the GCL (presumably more mature new neurons) from DCX-IR cells with horizontally orientated soma and dendrites in the SGZ or without dendritic projections [47]. We studied the ability of vitamin A supplementation to promote dendritic growth and neuronal maturation during aging. Quantitative analysis revealed that aged rats receiving a vitamin A enriched diet had significantly higher proportion of immature DCX-IR neurons with vertical dendrites, reflecting better maturation, compared to control aged rats [F(1,16) = 10.5, +12% between aged rats : 53.2±1.9% and enriched aged rats : 65.1±2.8%, p<0.01].

To investigate whether the higher degree of maturation of new born cells in enriched rats could be associated with a more pronounced survival of these cells, we analyzed cell survival using a 3-week interval between injection of BrdU and sacrifice of the animals. We found that most of the 3-week-old surviving BrdU-IR cells were isolated, round, large and located within the GCL (Fig. 4B). As shown in Fig. 4B, a vitamin A enriched diet from mid-life stimulated the survival BrdU-IR cells as they were more numerous in enriched-aged rats (+26%) than in their aged control group [F(1,16) = 8.5, number of BrdU-IR cells in aged rats : 793.3±65.7 vs in enriched aged rats : 1078±65.8, p<0.01].

The phenotype of newly born cells labeled with BrdU was determined using NeuN, a mature neuronal marker. The percentage of BrdU/NeuN double stained cells located in the GCL (Fig. 4C) was increased in enriched-aged rats [F(1,6) = 51.88, p<0.001, between aged rats: 14.4±1.7% and enriched-aged rats : 35.2±2.25%]. Thus, a more developped dendritic arborisation of immature neurons in enriched aged rats would result in a selective and better survival and neuronal differentiation of this particular population of newly born cells.

### Effects of a Mid-life vitamin A Supplementation on Retinoid Status in Aged Rats

#### Assay of serum retinol

**Figure 5:**
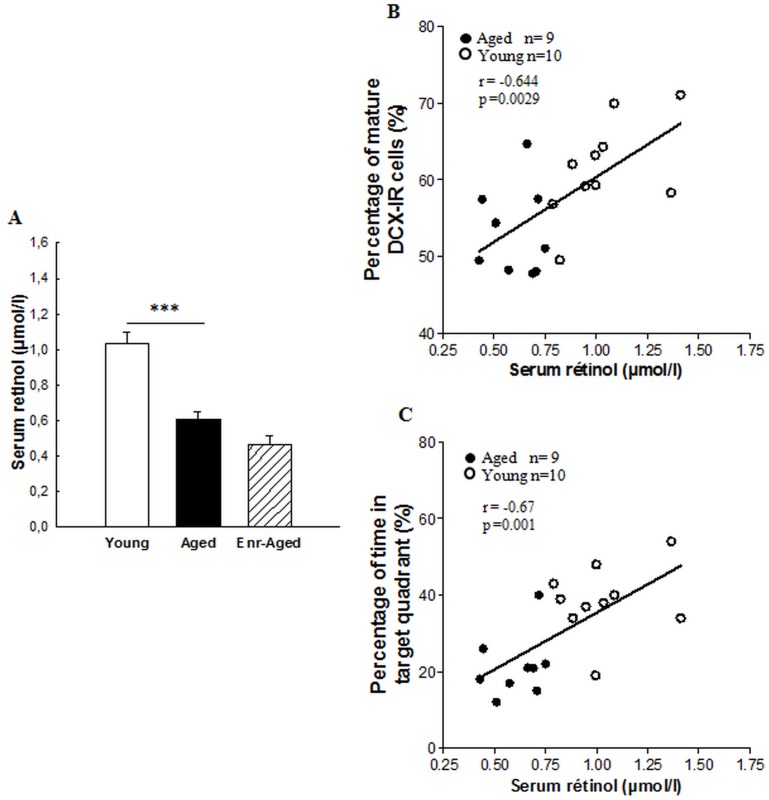
Effects of aging and a mid-life vitamin A supplementation on serum retinol levels. (A) Aging decreases serum retinol levels. Significant p values after Bonferroni correction for pairwise comparisons (level α = 0.025; ***p<0.001). (B)(C) Correlation analyses. (B) Percentage of mature DCX-IR cells and (C) spatial memory correlate with serum retinol levels in young and aged rats (r = 0.644, p<0.01 and r = 0.67, p<0.001 respectively).

The analysis of serum retinol levels was performed after the behavioral studies in order to confirm the retinoid status of aged rats (Fig. 5A). Quantitative analysis revealed differences between groups [F(2,25) = 28.72; p<0.001]. A significant reduction (- 41%) in serum retinol concentration was observed in aged rats relative to young rats (Bonferroni test, p<0.001, between young and aged rats; 1.033±0.06 µmol/l versus 0.6±0,04 µmol/l). However, a vitamin A supplementation diet did not increase serum retinol concentration in aged rats (Bonferroni test, n.s, between aged and enriched-aged rats; 0.6±0,04 µmol/l versus 0.465±0.05 µmol/l).

We examined the existence of linear correlations between the degree of maturation of newly born neurons and serum retinol levels for individual rats. A positive correlation was observed between the percentage of DCX-IR cells with vertical dendrites and serum retinol levels in young and aged rats (Fig. 5B: r = 0.64, p<0.002, n = 19) : rats with high level of serum retinol exhibited a higher percentage of DCX-IR cells with a more mature phenotype. Moreover, we have also observed a positive correlation between serum retinol levels and spatial memory in young and aged rats (Fig. 5C probe test and serum retinol level, r = 0.67, p = 0.001). No correlation has been observed if we integrate the enriched-aged group to the regression analysis (data not shown).

#### mRNA expression of CRABP-I in the hippocampus

**Figure 6:**
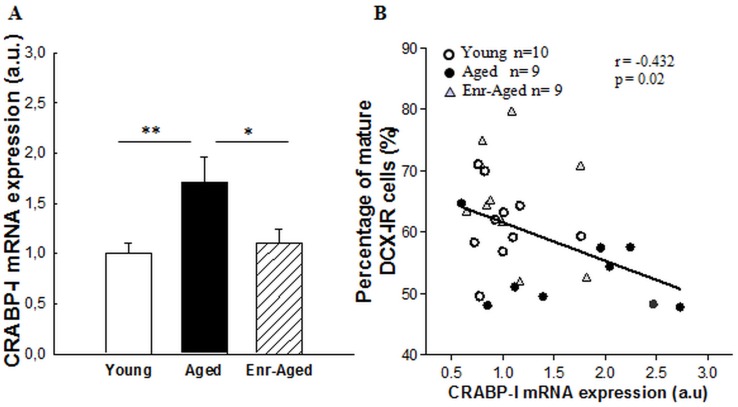
Effects of aging and a mid-life vitamin A supplementation on mRNA expression of CRABP-I in the hippocampus. (A) CRABP-I mRNA expression as quantified by Real Time-PCR. Vitamin A supplementation normalized aging-induced up-regulation of CRABP-I mRNA expresssion. Significant p values after Bonferroni correction for pairwise comparisons (level α = 0.025; *p<0.025). (B) Correlation analyses. Percentage of mature DCX-IR cells correlates with hippocampal CRABP-I mRNA expression in all groups (r = 0.43, p<0.05).

The ability of vitamin A supplementation to promote the maturation of newly generated neurons and improve memory abilities in aged rats could be in part mediated by the intracellular availability of RA which is determined by many regulatory proteins such as retinoic acid binding proteins I (CRABP-I), known to play an important role in RA-mediated differentiation processes [42]. As seen in Fig. 6A, quantitative analysis of hippocampal CRABP-I mRNA expression indicated differences between groups [F(2,25) = 4.95; p<0.025]. Indeed, we observed that aging up-regulates CRABP-I mRNA expression (+70%, Bonferroni test, p<0.01, between young rats : 1±0.09 a.u. and aged rats : 1.7±0.25 a.u.). By contrast, vitamin A supplementation reduced hippocampal CRABP-I mRNA expression in aged rats (Bonferroni test, p<0.025, between aged rats : 1.7±0.25 a.u. and enriched-aged rats : 1.1±0.13 a.u.).

We examined the existence of linear correlations between the degree of maturation of newly born neurons and hippocampal CRABP-I mRNA expression for the three groups of rats. A negative correlation was observed between CRABP-I mRNA expression levels and the percentage of DCX-IR cells with vertical dendrites (r = 0.432, p = 0.02, n = 28, Fig. 6B) : rats with lower level of hippocampal CRABP-I mRNA expression exhibited a high percentage of DCX-IR cells with vertical dendrites, reflecting a more mature phenotype. This correlation was also observed if we only integrated young and aged groups to the regression analysis (r = 0.476, p = 0.03, n = 19, data not shown).

## Discussion

In the present study, we demonstrate for the first time that 4 months of nutritional vitamin A supplementation started at mid-life improve the spatial memory and the level of differentiation of newborn neurons in aged Wistar rats. Moreover, our results suggest that hippocampal CRABP-I expression which controls the intracellular availability of RA, may be an important regulator of neuronal differentiation processes in the aged hippocampus.

In agreement with a previous study showing that a life-long vitamin A supplementation can prevent age-related short term working memory deterioration [15], our data confirm the idea that acting on the retinoid signalling pathway by a nutritional approach could be of great interest to alleviate age-induced memory deficits. Some memory deficits during aging can be partly explained by alterations of cerebral plasticity including adult hippocampal neurogenesis [48]. Moreover, age-related memory loss begins to appear at middle-age and coincides with reduced adult hippocampal neurogenesis [19], [49]. Thus, our study confirms the beneficial effects of vitamin A on age-induced spatial memory deficits but also demonstrates for the first time that supplementation started at mid-life is efficient to improve hippocampal neurogenesis and functions in aged rats.

Consistent with the age-related decline in hippocampal neurogenesis [19], [33], [50], [51], our study shows a strong decrease in cell proliferation and in the number of immature neurons in aging rats. Interestingly, we report that the spatial memory impairments in aged rats were accompanied by an abnormal differentiation of immature neurons. Here, we demonstrate that aged rats had significant lower mature DCX positive cells with vertical dendritic ramifications that are usually observed in young rats. This finding is in line with a recent study showing that age-related spatial memory impairments are associated with alterations in the differentiation of immature neurons [32]. Indeed, Nyffeler et al. have shown a higher proportion of Nestin-DCX double labelled cells in learning-impaired aged rats, suggesting a less mature stage of this pool of neuronal progenitors. This hypothesis is also in line with data showing a significant retardation of dendritic growth during the DCX expression phase of newborn neurons in aged rats [18].

Our experiments also suggest that promotion of maturation of newborn neurons in supplemented aged rats could be one plausible mechanism by which vitamin A supplementation might exert promnesic effects. Indeed, we have shown that vitamin A supplementation started at mid-life does not improve cell proliferation or the number of immature neurons in aged rats, but this preventive nutritional strategy is very potent to increase dendritic arborisation of DCX-positive cells, probably resulting in a better survival and neuronal differentiation. This is partly confirmed by the higher percentage of BrdU/NeuN cells observed in enriched-aged rats compared to aged rats. Thus, the integration of these newborn neurons into the hippocampal circuitry could facilitate spatial memory performances in the enriched-aged rats. Consistent with the present results, it has been shown that aged rats with preserved spatial memory exhibited a better survival and differentiation examined 3 weeks after learning (under basal conditions) compared to aged rats displaying spatial memory impairments [31]. Moreover, the ability to perform hippocampus-related functions could also result from changing the dynamics of hippocampal neurogenesis in the course of learning. Indeed, spatial learning has been reported to promote the survival of newborn cells generated before learning [52], [53], [54], to accelerate the differentiation of selected newborn cells toward a neuronal phenotype and to increase the dendritic development [55]. Thus, although our results might suggest that vitamin A supplementation could directly increase maturation of new neurons in aged rats showing better spatial memory performances, we cannot exclude the potential influence of spatial learning on hippocampal neurogenesis in the different groups.

Similarly, several studies using vitamin A deficiency (VAD) models, have shown that this vitamin is necessary for neuronal differentiation and survival [35] but also for cell proliferation in the DG of the hippocampus [36]. Indeed, it has been demonstrated that the effect of vitamin A deficient diet on the level of hippoccampal neurogenesis is reversible and that RA treatment can restore hippocampal neurogenesis and correct spatial memory deficits in vitamin A deficient rats [36]. Nutrition is an important extrinsic factor which modulates adult hippocampal neurogenesis [56], but other factors such as environmental enrichment or physical activity starting from middle-age, have also been shown to be efficient in the prevention of hippocampal neurogenesis and memory alterations [17], [47]. Indeed, comparable increases in BrdU-positive cells double-labeled for the neuronal marker NeuN have been described for aged mice stimulated with an enriched environment and showing preserved spatial learning performances [51].

Such effects on hippocampal neurogenesis may probably act in concert with stimulation of synaptic plasticity. Indeed, the suppression of neurogenesis by irradiation or genetic ablation has been shown to disrupt LTP in the dentate gyrus [57] and impairs some forms of hippocampus-dependent learning and memory [58], [59]. Conversely, conditions that stimulate adult neurogenesis in mice also increase LTP [60], [61]. Similarly, the activation of the RA signalling pathway can improve hippocampal plasticity either by enhancing synaptic strength as measured by LTP or by synaptic remodelling, known to modulate hippocampal-dependent memory processes [7], [8], [15], [62].

In order to better understand some potential mechanisms involved in the regulation of hippocampal neurogenesis and spatial learning by vitamin A supplementation, we have evaluated serum retinol concentrations and hippocampal CRABP-I expression, indicators of vitamin A status and intracellular availability of RA. Vitamin A is mobilized from liver stores and transported into the plasma bound to a carrier protein [63]. At the target cell level, retinol is oxidised to RA which binds to cellular retinoic acid-binding proteins (CRABPs). This binding is known to regulate the nuclear activity of RA [63], [64]. In the present study, aged rats exhibited a significant decrease in the serum retinol concentration compared to young rats, but vitamin A enriched diet failed to normalize this level. This reduction has been earlier reported in aged rats and humans [39], [40], [65] and could be explained during senescence by a loss of the capacity to mobilize vitamin A from the liver and thereby to regulate serum retinol levels, without affecting its ability of storage [66], [67]. Thus, these age-induced alterations of retinol metabolism would explain why the newly absorbed vitamin A from the diet was unable to correct serum retinol levels in aged rats. It has been shown that reduction in vitamin A metabolism during aging resulted in RA deficit at the cellular level [40]. However, in vitamin A deficiency, target tissues can directly use newly absorbed vitamin A from diet, in order to rapidly synthesize RA [41]. Thus, in enriched-aged rats which failed to normalize the serum retinol level, this newly absorbed vitamin A would be immediately used by target tissues such as the brain, to increase local synthesis of RA. In the rodent hippocampus, RA is synthesized from the meninges by anabolic enzymes (RALDHs) and would diffuse from this source to the blades of the DG [38]. It is probably one of the mechanisms by which vitamin A enriched diet would increase the levels of intracellular RA in the aged hippocampus without mobilization of vitamin A from the liver.

Moreover, the CRABPs (CRABP I and CRABP II) are also important regulators of the cellular bioavailability of RA in the brain [68], [69]. Only CRABP-I is expressed in the hippocampus and more particularly in the dendate gyrus [38]. The precise role of CRABPs in RA signalling is still unclear, but some data provide evidence that CRABP-I buffers the free intracellular RA, and regulates by this way, the amount accessible to its nuclear receptors and therefore, its activity [70], [71]. Here, we show that aging induced an up-regulation of hippocampal CRABP-I mRNA expression, normalized by vitamin A supplementation. This result suggests that vitamin A enriched-diet could normalize the level of free cellular RA in aged rats by a negative regulation of CRABP-I expression. This hypothesis is also sustained by a negative correlation evidenced between serum retinol levels and CRABP-I expression in young and aged groups (data not shown).

Together, the decreased hippocampal CRABP-I expression and the increased amount of intracellular RA synthesized from newly absorbed vitamin A in enriched aged group would lead to an over saturation of the binding capacities of CRABP-I. Therefore, the remaining free RA could bind to their nuclear receptors and modulate transcription of genes involved in hippocampal plasticity such as neurogenesis.

In the present study, we observed that inadequate vitamin A metabolism in aged rats could have some consequences on hippocampal neurogenesis and functions. Indeed, we evidenced a positive correlation between level of serum retinol, maturation of DCX positive cells and also spatial memory in young and aged groups. This result is coherent with other studies showing that demented patients exhibited low serum retinol concentration [72], [73]. In enriched aged rats, the newly absorbed vitamin A from diet would be used to increase local synthesis of RA which would stimulate hippocampal neurogenesis and spatial memory. This hypothesis is supported by the detection of a gradient of RA which diffuses from the meninges across the infrapyramidal and suprapyramidal blades of the DG to differentially regulate hippocampal neurogenesis between these two blades [38].

Moreover, it has been shown that CRABP-I could play a crucial role in differentiation processes *in vitro* : (i) the up-regulation of CRABP-I in human neuroblastoma cells reduced their differentiation potential [43], (ii) the overexpression of CRABP I in F9 cells decreased their RA-induced differentiation and high concentration of RA become required for this cells to recover the responsiveness to RA, (iii) a reduced level of CRABP I led to a higher RA sensitivity of the cells to differentiate [74], [75]. Accordingly to these studies, we have shown negative correlations between hippocampal CRABP-I expression and maturation of DCX positive cells suggesting that age-related up-regulation of hippocampal CRABP-I could contribute to hippocampal neurogenesis alterations. Moreover, this correlation is maintained if we integrate the enriched-aged group to the regression analysis. On the contrary, no significant correlations were found between serum retinol and hippocampal neurogenesis in all groups (young, aged and enriched-aged). Thus, even if during senescence there is a loss of the capacity to regulate serum retinol levels by the diet, the newly absorbed vitamin A would be immediately used by target tissues such as the hippocampus leading to the normalization of the level of CRABP-I gene expression which could be determinant for hippocampal differentiation processes and thereby for hippocampal functions.

Thus, the expression of CRABP-I gene must be tightly controlled in order to maintain a homeostatic supply of free intracellular RA but this bioavailability can also be evaluated by measuring the expression of retinoid receptors. Several studies have demonstrated age-related decreases in mRNA expression or protein levels of retinoid receptors in mice and rat brain [7], [11], [12], [15], [50] indicating a decreased bioavailability of the ligand, which can be normalized by RA treatment or vitamin A supplementation. Interestingly, it has recently been demonstrated that the dysfunctioning of retinoid signalling induced by VAD decreased immunoreactivity of some retinoid receptors (RARα and β) in the hippocampus, whereas immunoreactivity of RXRα and β was increased in the same area [76], [77]. Similarly, an hippocampal over-expression of RXRα has been found in the aged rats of the present study and this effect can be restored by vitamin A supplementation (data not shown). Thus, although the newly absorbed vitamin A from the diet was unable to correct serum retinol levels in aged rats, as previously discussed, vitamin A supplementation can modulate hippocampal expression of some retinoid receptors in aged rats suggesting that our findings on CRABP-I gene expression, neurogenesis and cognitive functions could be mediated, at least in part, through these retinoid receptors.

Altogether, the present study demonstrates for the first time that vitamin A supplementation from middle-age improves the level of differentiation of newly born neurons and enhances spatial memory during senescence. The normalization of the hippocampal expression of CRABP-I and thereby the increased bioavailability of intracellular RA could be a possible mechanism by which vitamin A supplementation acts on neuronal differentiation processes and exerts its promnesic effects during aging. As the dysfunctioning of retinoid signalling has been suggested to be involved in the aetiology of some neurodegenerative diseases such as Alzheimer’s Disease (AD) [78], [79], [80], [81], vitamin A supplementation from middle-age could be widely proposed for alleviating cognitive decline but also for delaying neurodegenerative processes.

## References

[1] McCaffery P, Drager UC (2000) Regulation of retinoic acid signaling in the embryonic nervous system: a master differentiation factor. Cytokine Growth Factor Rev 11: 233–249. 10817966 10.1016/s1359-6101(00)00002-2

[2] Maden M (2002) Retinoid signalling in the development of the central nervous system. Nat Rev Neurosci 3: 843–853. 12415292 10.1038/nrn963

[3] McCaffery PJ, Adams J, Maden M, Rosa-Molinar E (2003) Too much of a good thing: retinoic acid as an endogenous regulator of neural differentiation and exogenous teratogen. Eur J Neurosci 18: 457–472. 12911743 10.1046/j.1460-9568.2003.02765.x

[4] Lane MA, Bailey SJ (2005) Role of retinoid signalling in the adult brain. Prog Neurobiol 75: 275–293. 15882777 10.1016/j.pneurobio.2005.03.002

[5] McCaffery P, Zhang J, Crandall JE (2006) Retinoic acid signaling and function in the adult hippocampus. J Neurobiol 66: 780–791. 16688774 10.1002/neu.20237

[6] Olson CR, Mello CV (2010) Significance of vitamin A to brain function, behavior and learning. Mol Nutr Food Res 54: 489–495. 20077419 10.1002/mnfr.200900246 PMC3169332

[7] Etchamendy N, Enderlin V, Marighetto A, Vouimba RM, Pallet V, et al (2001) Alleviation of a selective age-related relational memory deficit in mice by pharmacologically induced normalization of brain retinoid signaling. J Neurosci 21: 6423–6429. 11487666 10.1523/JNEUROSCI.21-16-06423.2001 PMC6763177

[8] Misner DL, Jacobs S, Shimizu Y, de Urquiza AM, Solomin L, et al (2001) Vitamin A deprivation results in reversible loss of hippocampal long-term synaptic plasticity. Proc Natl Acad Sci U S A 98: 11714–11719. 11553775 10.1073/pnas.191369798 PMC58795

[9] Cocco S, Diaz G, Stancampiano R, Diana A, Carta M, et al (2002) Vitamin A deficiency produces spatial learning and memory impairment in rats. Neuroscience 115: 475–482. 12421614 10.1016/s0306-4522(02)00423-2

[10] Etchamendy N, Enderlin V, Marighetto A, Pallet V, Higueret P, et al (2003) Vitamin A deficiency and relational memory deficit in adult mice: relationships with changes in brain retinoid signalling. Behav Brain Res 145: 37–49. 14529804 10.1016/s0166-4328(03)00099-8

[11] Enderlin V, Pallet V, Alfos S, Dargelos E, Jaffard R, et al (1997) Age-related decreases in mRNA for brain nuclear receptors and target genes are reversed by retinoic acid treatment. Neurosci Lett 229: 125–129. 9223607 10.1016/s0304-3940(97)00424-2

[12] Feart C, Mingaud F, Enderlin V, Husson M, Alfos S, et al (2005) Differential effect of retinoic acid and triiodothyronine on the age-related hypo-expression of neurogranin in rat. Neurobiol Aging 26: 729–738. 15708448 10.1016/j.neurobiolaging.2004.06.004

[13] Eichenbaum H, Dudchenko P, Wood E, Shapiro M, Tanila H (1999) The hippocampus, memory, and place cells: is it spatial memory or a memory space? Neuron 23: 209–226. 10399928 10.1016/s0896-6273(00)80773-4

[14] Eichenbaum H (2004) Hippocampus: cognitive processes and neural representations that underlie declarative memory. Neuron 44: 109–120. 15450164 10.1016/j.neuron.2004.08.028

[15] Mingaud F, Mormede C, Etchamendy N, Mons N, Niedergang B, et al (2008) Retinoid hyposignaling contributes to aging-related decline in hippocampal function in short-term/working memory organization and long-term declarative memory encoding in mice. J Neurosci 28: 279–291. 18171945 10.1523/JNEUROSCI.4065-07.2008 PMC6671152

[16] Kuhn HG, Dickinson-Anson H, Gage FH (1996) Neurogenesis in the dentate gyrus of the adult rat: age-related decrease of neuronal progenitor proliferation. J Neurosci 16: 2027–2033. 8604047 10.1523/JNEUROSCI.16-06-02027.1996 PMC6578509

[17] Kempermann G, Gast D, Gage FH (2002) Neuroplasticity in old age: sustained fivefold induction of hippocampal neurogenesis by long-term environmental enrichment. Ann Neurol 52: 135–143. 12210782 10.1002/ana.10262

[18] Rao MS, Hattiangady B, Abdel-Rahman A, Stanley DP, Shetty AK (2005) Newly born cells in the ageing dentate gyrus display normal migration, survival and neuronal fate choice but endure retarded early maturation. Eur J Neurosci 21: 464–476. 15673445 10.1111/j.1460-9568.2005.03853.x

[19] Driscoll I, Howard SR, Stone JC, Monfils MH, Tomanek B, et al (2006) The aging hippocampus: a multi-level analysis in the rat. Neuroscience 139: 1173–1185. 16564634 10.1016/j.neuroscience.2006.01.040

[20] Drapeau E, Nora Abrous D (2008) Stem cell review series: role of neurogenesis in age-related memory disorders. Aging Cell 7: 569–589. 18221417 10.1111/j.1474-9726.2008.00369.x PMC2990912

[21] Gross CG (2000) Neurogenesis in the adult brain: death of a dogma. Nat Rev Neurosci 1: 67–73. 11252770 10.1038/35036235

[22] Cameron HA, Woolley CS, McEwen BS, Gould E (1993) Differentiation of newly born neurons and glia in the dentate gyrus of the adult rat. Neuroscience 56: 337–344. 8247264 10.1016/0306-4522(93)90335-d

[23] Piatti VC, Esposito MS, Schinder AF (2006) The timing of neuronal development in adult hippocampal neurogenesis. Neuroscientist 12: 463–468. 17079512 10.1177/1073858406293538

[24] Abrous DN, Koehl M, Le Moal M (2005) Adult neurogenesis: from precursors to network and physiology. Physiol Rev 85: 523–569. 15788705 10.1152/physrev.00055.2003

[25] Leuner B, Gould E, Shors TJ (2006) Is there a link between adult neurogenesis and learning? Hippocampus 16: 216–224. 16421862 10.1002/hipo.20153

[26] Deng W, Aimone JB, Gage FH (2010) New neurons and new memories: how does adult hippocampal neurogenesis affect learning and memory? Nat Rev Neurosci 11: 339–350. 20354534 10.1038/nrn2822 PMC2886712

[27] Dupret D, Revest JM, Koehl M, Ichas F, De Giorgi F, et al (2008) Spatial relational memory requires hippocampal adult neurogenesis. PLoS One 3: e1959. 18509506 10.1371/journal.pone.0001959 PMC2396793

[28] Snyder JS, Hong NS, McDonald RJ, Wojtowicz JM (2005) A role for adult neurogenesis in spatial long-term memory. Neuroscience 130: 843–852. 15652983 10.1016/j.neuroscience.2004.10.009

[29] Zhang CL, Zou Y, He W, Gage FH, Evans RM (2008) A role for adult TLX-positive neural stem cells in learning and behaviour. Nature 451: 1004–1007. 18235445 10.1038/nature06562

[30] Garthe A, Behr J, Kempermann G (2009) Adult-generated hippocampal neurons allow the flexible use of spatially precise learning strategies. PLoS One 4: e5464. 19421325 10.1371/journal.pone.0005464 PMC2674212

[31] Drapeau E, Mayo W, Aurousseau C, Le Moal M, Piazza PV, et al (2003) Spatial memory performances of aged rats in the water maze predict levels of hippocampal neurogenesis. Proc Natl Acad Sci U S A 100: 14385–14390. 14614143 10.1073/pnas.2334169100 PMC283601

[32] Nyffeler M, Yee BK, Feldon J, Knuesel I (2010) Abnormal differentiation of newborn granule cells in age-related working memory impairments. Neurobiol Aging 31: 1956–1974. 19100662 10.1016/j.neurobiolaging.2008.10.014

[33] Bizon JL, Gallagher M (2003) Production of new cells in the rat dentate gyrus over the lifespan: relation to cognitive decline. Eur J Neurosci 18: 215–219. 12859354 10.1046/j.1460-9568.2003.02733.x

[34] Bizon JL, Lee HJ, Gallagher M (2004) Neurogenesis in a rat model of age-related cognitive decline. Aging Cell 3: 227–234. 15268756 10.1111/j.1474-9728.2004.00099.x

[35] Jacobs S, Lie DC, DeCicco KL, Shi Y, DeLuca LM, et al (2006) Retinoic acid is required early during adult neurogenesis in the dentate gyrus. Proc Natl Acad Sci U S A 103: 3902–3907. 16505366 10.1073/pnas.0511294103 PMC1450163

[36] Bonnet E, Touyarot K, Alfos S, Pallet V, Higueret P, et al (2008) Retinoic acid restores adult hippocampal neurogenesis and reverses spatial memory deficit in vitamin A deprived rats. PLoS One 3: e3487. 18941534 10.1371/journal.pone.0003487 PMC2567033

[37] Crandall J, Sakai Y, Zhang J, Koul O, Mineur Y, et al (2004) 13-cis-retinoic acid suppresses hippocampal cell division and hippocampal-dependent learning in mice. Proc Natl Acad Sci U S A 101: 5111–5116. 15051884 10.1073/pnas.0306336101 PMC387382

[38] Goodman T, Crandall JE, Nanescu SE, Quadro L, Shearer K, et al.. (2012) Patterning of retinoic acid signaling and cell proliferation in the hippocampus. Hippocampus. 10.1002/hipo.22037 PMC3505796 22689466

[39] van der Loo B, Labugger R, Aebischer CP, Bachschmid M, Spitzer V, et al (2004) Age-related changes of vitamin A status. J Cardiovasc Pharmacol 43: 26–30. 14668564 10.1097/00005344-200401000-00005

[40] Feart C, Pallet V, Boucheron C, Higueret D, Alfos S, et al (2005) Aging affects the retinoic acid and the triiodothyronine nuclear receptor mRNA expression in human peripheral blood mononuclear cells. Eur J Endocrinol 152: 449–458. 15757863 10.1530/eje.1.01858

[41] Ross AC, Russell RM, Miller SA, Munro IC, Rodricks JV, et al (2009) Application of a key events dose-response analysis to nutrients: a case study with vitamin A (retinol). Crit Rev Food Sci Nutr 49: 708–717. 19690996 10.1080/10408390903098749 PMC2840874

[42] Wilson LJ, Myat A, Sharma A, Maden M, Wingate RJ (2007) Retinoic acid is a potential dorsalising signal in the late embryonic chick hindbrain. BMC Dev Biol 7: 138. 18093305 10.1186/1471-213X-7-138 PMC2266733

[43] Uhrig M, Brechlin P, Jahn O, Knyazev Y, Weninger A, et al (2008) Upregulation of CRABP1 in human neuroblastoma cells overproducing the Alzheimer-typical Abeta42 reduces their differentiation potential. BMC Med 6: 38. 19087254 10.1186/1741-7015-6-38 PMC2645429

[44] Penniston KL, Tanumihardjo SA (2006) The acute and chronic toxic effects of vitamin A. Am J Clin Nutr. 83: 191–201. 10.1093/ajcn/83.2.191 16469975

[45] Lemaire V, Lamarque S, Le Moal M, Piazza PV, Abrous DN (2006) Postnatal stimulation of the pups counteracts prenatal stress-induced deficits in hippocampal neurogenesis. Biol Psychiatry 59: 786–792. 16460692 10.1016/j.biopsych.2005.11.009

[46] Biesalski HK, Ehrenthal W, Gross M, Hafner G, Harth O (1983) Rapid determination of retinol (vitamin A) in serum by high pressure liquid chromatography (HPLC). Int J Vitam Nutr Res 53: 130–137. 6885272

[47] Marlatt MW, Potter MC, Lucassen PJ, van Praag H (2012) Running throughout middle-age improves memory function, hippocampal neurogenesis, and BDNF levels in female C57BL/6J mice. Dev Neurobiol 72: 943–952. 22252978 10.1002/dneu.22009 PMC3485396

[48] Koehl M, Abrous DN (2011) A new chapter in the field of memory: adult hippocampal neurogenesis. Eur J Neurosci 33: 1101–1114. 21395854 10.1111/j.1460-9568.2011.07609.x

[49] Erickson CA, Barnes CA (2003) The neurobiology of memory changes in normal aging. Exp Gerontol 38: 61–69. 12543262 10.1016/s0531-5565(02)00160-2

[50] Dyall SC, Michael GJ, Michael-Titus AT (2010) Omega-3 fatty acids reverse age-related decreases in nuclear receptors and increase neurogenesis in old rats. J Neurosci Res 88: 2091–2102. 20336774 10.1002/jnr.22390

[51] Kempermann G, Kuhn HG, Gage FH (1998) Experience-induced neurogenesis in the senescent dentate gyrus. J Neurosci 18: 3206–3212. 9547229 10.1523/JNEUROSCI.18-09-03206.1998 PMC6792643

[52] Drapeau E, Montaron MF, Aguerre S, Abrous DN (2007) Learning-induced survival of new neurons depends on the cognitive status of aged rats. J Neurosci 27: 6037–6044. 17537975 10.1523/JNEUROSCI.1031-07.2007 PMC6672254

[53] Gould E, Beylin A, Tanapat P, Reeves A, Shors TJ (1999) Learning enhances adult neurogenesis in the hippocampal formation. Nat Neurosci 2: 260–265. 10195219 10.1038/6365

[54] Dupret D, Fabre A, Dobrossy MD, Panatier A, Rodriguez JJ, et al (2007) Spatial learning depends on both the addition and removal of new hippocampal neurons. PLoS Biol 5: e214. 17683201 10.1371/journal.pbio.0050214 PMC1939885

[55] Tronel S, Fabre A, Charrier V, Oliet SH, Gage FH, et al (2010) Spatial learning sculpts the dendritic arbor of adult-born hippocampal neurons. Proc Natl Acad Sci U S A 107: 7963–7968. 20375283 10.1073/pnas.0914613107 PMC2867872

[56] Zainuddin MS, Thuret S (2012) Nutrition, adult hippocampal neurogenesis and mental health. Br Med Bull 103: 89–114. 22833570 10.1093/bmb/lds021

[57] Singer BH, Gamelli AE, Fuller CL, Temme SJ, Parent JM, et al (2011) Compensatory network changes in the dentate gyrus restore long-term potentiation following ablation of neurogenesis in young-adult mice. Proc Natl Acad Sci U S A 108: 5437–5442. 21402918 10.1073/pnas.1015425108 PMC3069161

[58] Shimazu K, Zhao M, Sakata K, Akbarian S, Bates B, et al (2006) NT-3 facilitates hippocampal plasticity and learning and memory by regulating neurogenesis. Learn Mem 13: 307–315. 16705139 10.1101/lm.76006 PMC1475811

[59] Saxe MD, Battaglia F, Wang JW, Malleret G, David DJ, et al (2006) Ablation of hippocampal neurogenesis impairs contextual fear conditioning and synaptic plasticity in the dentate gyrus. Proc Natl Acad Sci U S A 103: 17501–17506. 17088541 10.1073/pnas.0607207103 PMC1859958

[60] van Praag H, Christie BR, Sejnowski TJ, Gage FH (1999) Running enhances neurogenesis, learning, and long-term potentiation in mice. Proc Natl Acad Sci U S A 96: 13427–13431. 10557337 10.1073/pnas.96.23.13427 PMC23964

[61] Pujadas L, Gruart A, Bosch C, Delgado L, Teixeira CM, et al (2010) Reelin regulates postnatal neurogenesis and enhances spine hypertrophy and long-term potentiation. J Neurosci 30: 4636–4649. 20357114 10.1523/JNEUROSCI.5284-09.2010 PMC6632327

[62] Chiang MY, Misner D, Kempermann G, Schikorski T, Giguere V, et al (1998) An essential role for retinoid receptors RARbeta and RXRgamma in long-term potentiation and depression. Neuron 21: 1353–1361. 9883728 10.1016/s0896-6273(00)80654-6

[63] Blomhoff R (1994) Transport and metabolism of vitamin A. Nutr Rev. 52: S13–23. 10.1111/j.1753-4887.1994.tb01382.x 8202278

[64] Zhang YR, Zhao YQ, Huang JF (2012) Retinoid-binding proteins: similar protein architectures bind similar ligands via completely different ways. PLoS One 7: e36772. 22574224 10.1371/journal.pone.0036772 PMC3344936

[65] Haller J, Weggemans RM, Lammi-Keefe CJ, Ferry M (1996) Changes in the vitamin status of elderly Europeans: plasma vitamins A, E, B-6, B-12, folic acid and carotenoids. SENECA Investigators. Eur J Clin Nutr 50 Suppl 2S32–46. 8841783

[66] Borel P, Mekki N, Boirie Y, Partier A, Alexandre-Gouabau MC, et al (1998) Comparison of the postprandial plasma vitamin A response in young and older adults. J Gerontol A Biol Sci Med Sci 53: B133–140. 9520909 10.1093/gerona/53a.2.b133

[67] Azais-Braesco V, Dodeman I, Delpal S, Alexandre-Gouabau MC, Partier A, et al (1995) Vitamin A contained in the lipid droplets of rat liver stellate cells is substrate for acid retinyl ester hydrolase. Biochim Biophys Acta 1259: 271–276. 8541334 10.1016/0005-2760(95)00173-5

[68] Ong DE (1994) Cellular transport and metabolism of vitamin A: roles of the cellular retinoid-binding proteins. Nutr Rev 52: S24–31. 10.1111/j.1753-4887.1994.tb01383.x 8202279

[69] Giguere V, Evans RM (1990) Identification of receptors for retinoids as members of the steroid and thyroid hormone receptor family. Methods Enzymol 189: 223–232. 1963457 10.1016/0076-6879(90)89293-q

[70] Maden M, Ong DE, Summerbell D, Chytil F (1988) Spatial distribution of cellular protein binding to retinoic acid in the chick limb bud. Nature 335: 733–735. 2845280 10.1038/335733a0

[71] Perez-Castro AV, Toth-Rogler LE, Wei LN, Nguyen-Huu MC (1989) Spatial and temporal pattern of expression of the cellular retinoic acid-binding protein and the cellular retinol-binding protein during mouse embryogenesis. Proc Natl Acad Sci U S A 86: 8813–8817. 2554331 10.1073/pnas.86.22.8813 PMC298380

[72] Polidori MC (2004) Oxidative stress and risk factors for Alzheimer’s disease: clues to prevention and therapy. J Alzheimers Dis 6: 185–191. 15096703 10.3233/jad-2004-6211

[73] Rinaldi P, Polidori MC, Metastasio A, Mariani E, Mattioli P, et al (2003) Plasma antioxidants are similarly depleted in mild cognitive impairment and in Alzheimer’s disease. Neurobiol Aging 24: 915–919. 12928050 10.1016/s0197-4580(03)00031-9

[74] Boylan JF, Gudas LJ (1991) Overexpression of the cellular retinoic acid binding protein-I (CRABP-I) results in a reduction in differentiation-specific gene expression in F9 teratocarcinoma cells. J Cell Biol 112: 965–979. 1847931 10.1083/jcb.112.5.965 PMC2288868

[75] Boylan JF, Gudas LJ (1992) The level of CRABP-I expression influences the amounts and types of all-trans-retinoic acid metabolites in F9 teratocarcinoma stem cells. J Biol Chem 267: 21486–21491. 1328234

[76] Arfaoui A, Nasri I, Boulbaroud S, Ouichou A, Mesfioui A (2009) Effect of vitamin A deficiency on retinol and retinyl esters contents in rat brain. Pak J Biol Sci 12: 939–948. 19817120 10.3923/pjbs.2009.939.948

[77] Arfaoui A, Lobo MV, Boulbaroud S, Ouichou A, Mesfioui A, et al.. (2012) Expression of retinoic acid receptors and retinoid X receptors in normal and vitamin A deficient adult rat brain. Ann Anat. 10.1016/j.aanat.2012.06.006 23017197

[78] Goodman AB, Pardee AB (2003) Evidence for defective retinoid transport and function in late onset Alzheimer’s disease. Proc Natl Acad Sci U S A 100: 2901–2905. 12604774 10.1073/pnas.0437937100 PMC151438

[79] Goodman AB (2006) Retinoid receptors, transporters, and metabolizers as therapeutic targets in late onset Alzheimer disease. J Cell Physiol 209: 598–603. 17001693 10.1002/jcp.20784

[80] Corcoran JP, So PL, Maden M (2004) Disruption of the retinoid signalling pathway causes a deposition of amyloid beta in the adult rat brain. Eur J Neurosci 20: 896–902. 15305858 10.1111/j.1460-9568.2004.03563.x

[81] Husson M, Enderlin V, Delacourte A, Ghenimi N, Alfos S, et al (2006) Retinoic acid normalizes nuclear receptor mediated hypo-expression of proteins involved in beta-amyloid deposits in the cerebral cortex of vitamin A deprived rats. Neurobiol Dis 23: 1–10. 16531051 10.1016/j.nbd.2006.01.008

